# Digitally Guided Hybrid Maxillary Expansion with Supragingival Mandibular Miniplates for Class III Correction in Late Adolescents: A Pilot Clinical Study

**DOI:** 10.3390/jcm15083070

**Published:** 2026-04-17

**Authors:** Ignasi Arcos, Andre Walter, Théophile Marc, Nuria Clusellas, Andreu Puigdollers

**Affiliations:** Facultat d’Odontologia, Departament d’Ortodoncia, Universitat Internacional de Catalunya, C/Josep Trueta s/n, 08195 Barcelona, Spainnclusellas@uic.es (N.C.);

**Keywords:** Class III malocclusion, maxillary protraction, skeletal anchorage, hybrid maxillary expander, mandibular miniplates, digital workflow, orthodontic miniscrews, late adolescence, Alt-RAMEC protocol, pilot clinical study

## Abstract

**Background:** Management of skeletal Class III malocclusion of maxillary origin in late adolescence remains challenging, as conventional tooth-borne orthopedic approaches show reduced effectiveness at advanced stages of skeletal maturation. Minimally invasive, bone-anchored alternatives supported by digital workflows may improve clinical feasibility and patient acceptance. **Objective:** To describe a digitally guided clinical protocol combining a hybrid maxillary expander and supragingival mandibular miniplates, and to explore skeletal and dentoalveolar outcomes in late adolescents. **Methods:** This retrospective pilot clinical study included ten late adolescents (mean age 16.0 ± 1.3 years; range 13.8–17.7) in advanced skeletal maturation stages (CS4–CS6) with skeletal Class III malocclusion of maxillary origin. Treatment consisted of a hybrid maxillary expander anchored to palatal miniscrews and custom supragingival mandibular miniplates, placed using a fully digital workflow. Maxillary protraction was performed using a modified Alt-RAMEC protocol followed by continuous intermaxillary elastic traction for 12 months. Pre- and post-treatment cephalometric analyses were conducted. **Results:** A significant increase in SNA was observed (mean +6.1°, *p* < 0.001), indicating forward maxillary displacement. The Wits appraisal improved by 3.3 mm (*p* = 0.007), and the SeMax increased by 2.9 mm (*p* = 0.0013). No significant changes were found in the SNB or mandibular plane angle. Dentoalveolar effects were limited. **Conclusions:** Within the limitations of this pilot clinical study, the proposed digitally guided protocol demonstrated clinically relevant maxillary advancement with minimal dentoalveolar side effects and preserved vertical control. This relatively minimally invasive approach compared to conventional subgingival miniplates and orthognathic surgery may represent a feasible treatment option for selected late adolescent patients. Further controlled studies are required to confirm these findings.

## 1. Introduction

Skeletal Class III malocclusion is characterized by an anteroposterior discrepancy between the maxilla and mandible, arising from maxillary deficiency, mandibular prognathism, or a combination of both. Clinically, it commonly presents with an anterior crossbite and a concave facial profile, adversely affecting facial esthetics, occlusion, and oral function [1].

Historically, young patients with maxillary hypoplasia have been treated with facemask therapy delivering approximately 400–450 g of anterior traction to stimulate maxillary growth and redirect mandibular development [2,3]. Although forward and downward maxillary displacement with a favorable mandibular growth direction has been reported [2,4], such protocols frequently cause posterior mandibular rotation, increased lower facial height, and dentoalveolar compensations—upper incisor proclination and lower incisor uprighting—together with a strong dependence on patient compliance, which rarely exceeds 12–14 h/day in practice [2,5,6,7,8].

The advent of skeletal anchorage systems represents a major advance in contemporary orthodontics [9]. These approaches enable the management of complex malocclusions—previously requiring extractions or orthognathic surgery—via minimally invasive biomechanics and enhanced control and efficiency with reduced biological and financial costs [10]. Among these innovations, titanium miniplates introduced by De Clerck [11] permit near-continuous orthopedic force application between the maxilla and mandible, while minimizing dentoalveolar side effects associated with tooth-borne appliances; the plates are surgically inserted and positioned subgingivally to provide stable skeletal anchorage and direct force transmission to bone.

Several orthopedic strategies for Class III correction have been described [12,13,14]. However, effective maxillary protraction in late adolescence is largely restricted to bone-anchored approaches [15,16]. Late adolescence represents a transitional period in which conventional orthopedic approaches often show reduced effectiveness, while surgical correction may not yet be indicated or accepted by patients.

Studies have shown that intermaxillary elastics attached to miniplates can produce significant skeletal effects in patients aged 10–13 years, particularly when treatment starts in late mixed or early permanent dentition [17].

In parallel, digital technology has become integral to modern orthodontics. Intraoral scanning produces accurate STL files that can be merged with CBCT (DICOM) and stereophotogrammetry (OBJ) to build a comprehensive three-dimensional virtual patient [18]. This workflow enhances diagnostic precision and enables more predictive, individualized planning [19].

In this protocol, the supragingival miniplate concept refers specifically to the mandibular anchorage system, while maxillary protraction is supported by a digitally planned bone-anchored hybrid expander. Accordingly, the aim of this pilot clinical study was to describe a digitally guided protocol for Class III correction by using supragingival mandibular miniplates and a hybrid maxillary expander and to explore its clinical and skeletal outcomes. In this context, late adolescence refers to patients in advanced skeletal maturation stages (CS4–CS6), regardless of chronological age.

## 2. Materials and Methods

Ethics approval was obtained from the Ethics and Drug Research Committee of Universitat Internacional de Catalunya, under protocol ORT-ECL-2025-03.

In this retrospective pilot clinical study, ten late adolescents (five males, five females; mean age 16.0 ± 1.3 years; range 13.8–17.7) with skeletal Class III malocclusion of maxillary origin and a negative overjet ranging from −1 to −6 mm were included. Late adolescence was defined based on skeletal maturation stages (CS4–CS6), regardless of chronological age. This design was adopted because all patients had already been treated using the described protocol prior to data collection, and no prospective intervention was performed for research purposes.

The protocol sought to correct the skeletal Class III relationship using skeletal anchorage: a hybrid maxillary expander with hooks on molar bands for an optional facemask attachment, and custom supragingival mandibular plates supporting interarch Class III elastics.

A total of 60 self-drilling titanium miniscrews (2.0 mm diameter, 7–13 mm length; Swift model, Microdent System, Santa Eulàlia de Ronçana, Barcelona, Spain) were placed—two in the maxilla to anchor the hybrid expander and four in the mandible (two per buccal side). The use of two miniscrews per side in the mandible allowed for improved mechanical stability, enhanced load distribution, and reduced rotational forces on the supragingival plates during continuous orthopedic traction, thereby increasing the reliability of skeletal anchorage.

The inclusion criteria were:

(1) Skeletal Class III malocclusion of maxillary origin.
(2) Cervical vertebral maturation stages CS4–CS6.
(3) Negative overjet between −1 and −6 mm.
(4) Adequate bone density (D1–D2 according to Misch classification).
(5) Sufficient palatal and mandibular bone volume for miniscrew placement.

All patients presented with permanent dentition, no previous orthopedic Class III treatment, good oral hygiene, and absence of active periodontal disease or untreated caries at baseline.

The exclusion criteria were:

(1) Negative overjet greater than −6 mm.
(2) Poor bone density (D3–D5).
(3) Inadequate bone volume (palatal height < 6 mm or mandibular depth < 7 mm).
(4) Systemic disease or medication affecting bone metabolism (e.g., NSAIDs).

All miniscrews were inserted by using a digitally guided protocol to optimize accuracy, parallelism, and anatomical safety.

### 2.1. Digital Planning and Miniscrew Insertion Sites

**Figure 1:**
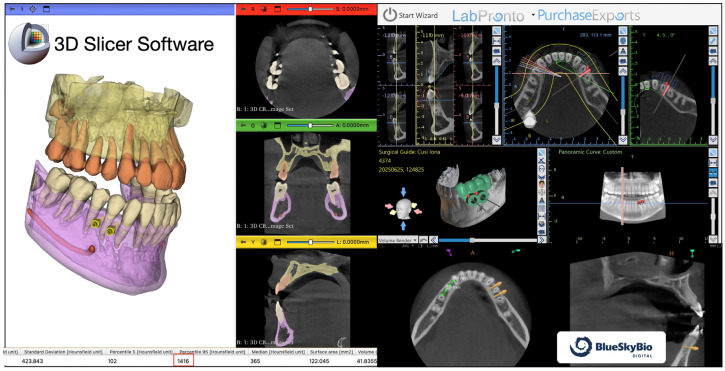
3D slicer software used to help measure the bone density area and Blue Sky Bio software to help the placement of the miniscrews.

Potential insertion sites were evaluated to confirm adequate bone density using a 3D Slicer (version 5.2.x; Brigham and Women’s Hospital, Boston, MA, USA; available at https://www.slicer.org; accessed on 1 March 2026), which allows for approximate quantification of Hounsfield units (HU) in selected regions (Figure 1). The spatial positioning of miniscrews was digitally planned in the Blue Sky Plan (version 4.13.x; Blue Sky Bio, Libertyville, IL, USA; available at https://www.blueskybio.com; accessed on 1 March 2026) for both arches (Figure 1).

Two data sets were required: DICOM CBCT (Carestream 3200, Kodak, Germany) and STL intraoral scans (3Shape TRIOS, Copenhagen, Denmark). The datasets were aligned for accurate anatomic correlation.

Maxillary miniscrews were placed in the M4 region as per Winsauer [20]—midway between the midpalatal suture and first premolar—ensuring the bicortical engagement of the palatal and nasal fossa cortices. Mandibular miniscrews (four per patient) were inserted interradicularly between 33–34, 34–35, 43–44, and 44–45, at least 1 mm apical to the mucogingival junction, with bicortical anchorage from buccal to lingual cortices and with an approximate 20° apical angulation as a general guideline, which was individually adapted based on CBCT-guided digital planning, according to each patient’s anatomical conditions. All sites met D1–D2 density criteria.

**Figure 2:**
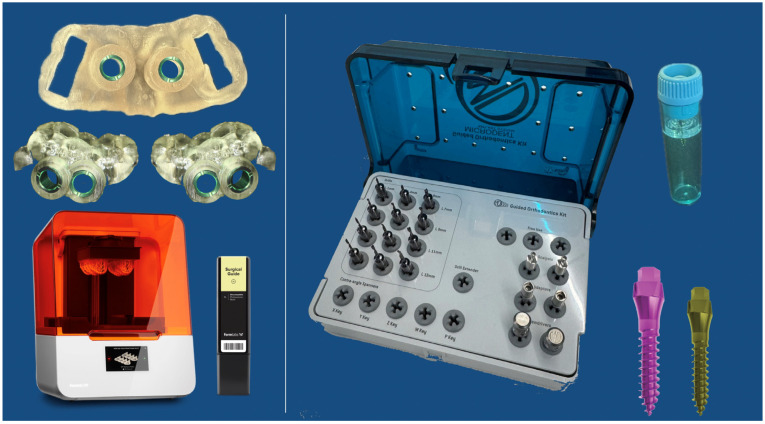
Three individual surgical guides printed with the adequate technology were designed for each patient. A Microdent Swift miniscrew and Microdent Orthodontic guided surgical kit (Microdent System, Barcelona, Spain) were used.

Three custom surgical guides were designed for each patient in the Blue Sky Plan: one maxillary and two mandibular (one per side; Figure 2). The mandibular guides included a bucco-occlusal notch to facilitate atraumatic sectioning and removal after placement, preserving the primary stability. Guides were printed on a Formlabs 3B (Formlabs, Somerville, MA, USA) with Surgical Guide Resin per validated protocols.

### 2.2. Guided Insertion Protocol for Orthodontic Miniscrews

All miniscrews were placed with the printed surgical guides. For D1–D2 bone—used in this cohort—predrilling was performed at 500 rpm with a surgical motor (W&H ImplantMed plus SI-1023, Bürmoos, Austria), using a pilot drill that was 0.6 mm narrower and 2 mm shorter than the selected screw. For example, a 2.0 × 11 mm screw used a 1.4 × 9 mm drill from the Microdent guided kit (Microdent System, Barcelona, Spain) (Figure 2). The undersized protocol enhanced the primary stability, particularly in the final 2 mm of insertion, engaging the nasal or lingual cortical plate for bicortical anchorage. Predrilling reduced the insertion torque, preventing values > 35 Ncm that may compromise stability or increase fracture risk [21]. The recommended torque for D1–D2 bone was 25–35 Ncm [22]; 2 mm screws fracture at 41–46 Ncm and 1.5–1.6 mm screws at 21–25 Ncm [22].

For D3 bone, only the cortex was perforated, to lower the torque and preserve medullary integrity (optimal torque 10–25 Ncm) [23,24]. For D4 bone, no drilling was performed; stability relied on mechanical engagement (5–10 Ncm) [24].

After predrilling, miniscrews were inserted bicortically at 25–35 Ncm using the Microdent screw carrier (Swift compatible). The surgical guide was sectioned occlusally and removed in two parts to avoid disturbing stability.

**Figure 3:**
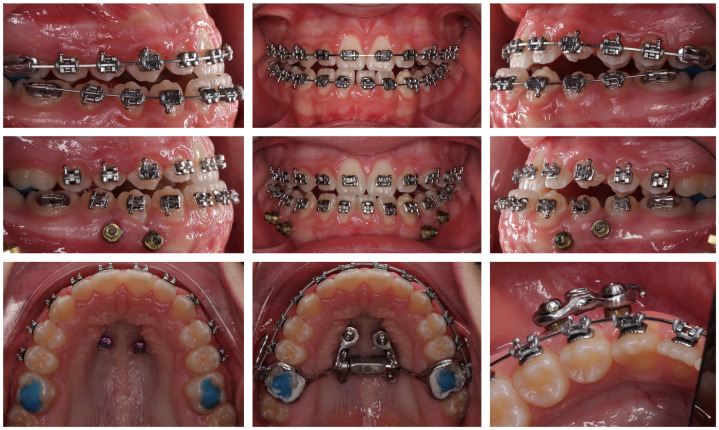
Maxillary miniscrews were positioned to help to support a hybrid expander. Mandibular miniscrews were positioned interradicularly between teeth 33–34, 34–35, 43–44, and 44–45, at least 1 mm apical to the mucogingival junction, achieving bicortical anchorage from the buccal to the lingual cortical plates.

**Figure 4:**
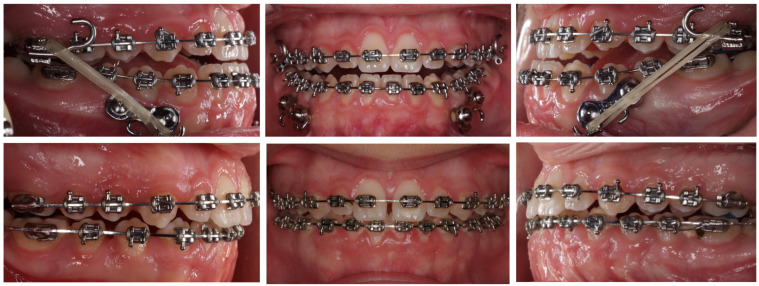
Hybrid maxillary expander anchored to both miniscrews and the first molars for the upper arch. The device incorporated a Power Screw expansion screw (Tiger Dental GmbH, Bregenz, Austria), with hooks soldered to the molar bands for subsequent Class III elastic attachment. For the mandibular arch, supragingival plates were custom-fabricated and secured to the miniscrews using fixation screws.

Both arches were scanned with miniscrews in situ, including bilateral bite registrations. Digital records were sent to a laboratory for the fabrication of a hybrid maxillary expander anchored to miniscrews and first molars (Power Screw–Tiger Dental GmbH, Bregenz, Austria) with hooks for elastic attachment. Custom mandibular supragingival plates were fabricated and fixed to the miniscrews with fixation screws (Figure 3 and Figure 4).

### 2.3. Maxillary Protraction with the Modified Alt-RAMEC Protocol (ArW-Alt-RAMEC)

Patients underwent a modified Alt-RAMEC sequence based on previously described protocols [25,26,27], which have demonstrated effectiveness in enhancing maxillary protraction by promoting circummaxillary suture mobilization. The protocol consisted of one week of daily activation followed by one week of deactivation per cycle, repeated for six weeks (three cycles). Thereafter, the expander was activated once per day until adequate expansion was achieved (mean 5.2 weeks).

**Figure 5:**
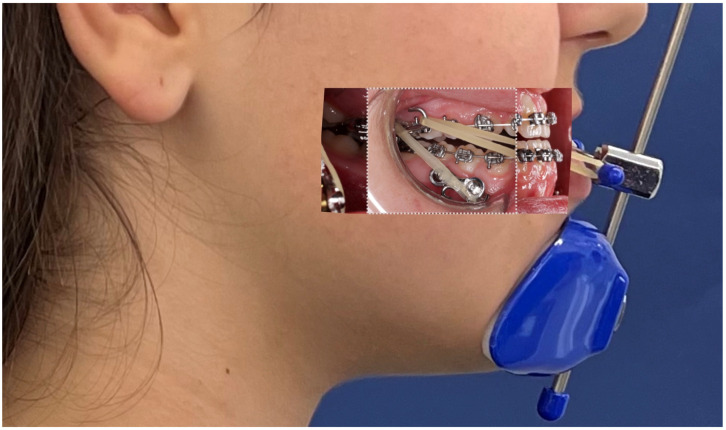
Additionally, 14-ounce, 5/16-inch elastics were used at night only, connecting the traction hooks to the facemask.

Orthopedic forces were applied with 8-oz, 3/16-inch elastics (American Orthodontics, Sheboygan, WI, USA), worn 24 h/day from mandibular plates to upper molar hooks. At night, additional 14-oz, 5/16-inch elastics connected traction hooks to a facemask based on the modifications of the Petit facemask derived from the original Delair concept [28] (Figure 5). The elastics were replaced daily. The total protraction lasted 12 months. Intermaxillary Class III elastics between the mandibular supragingival plates and the maxillary hybrid expander constituted the primary orthopedic force system, while nighttime facemask use was employed as an adjunctive support.

### 2.4. Treatment Adherence

Patient compliance with intermaxillary elastics and facemask use was monitored at each follow-up visit through patient self-reporting and clinical evaluation of elastic wear and appliance condition. Although no objective compliance devices were used, all patients reported consistent adherence to the prescribed protocol.

### 2.5. Cephalometric Evaluation

Skeletal changes were assessed via Steiner analysis (SNA, SNB, ANB), mandibular plane angle (SN–GoGn), and incisor position/inclination (U1, L1). Sagittal relationships were evaluated with the Wits appraisal. Skeletal maturation was determined with cervical vertebral maturation (CVM).

### 2.6. Maxillary Effective Protraction (SeMax Line): A Novel Linear Cephalometric Reference

**Figure 6:**
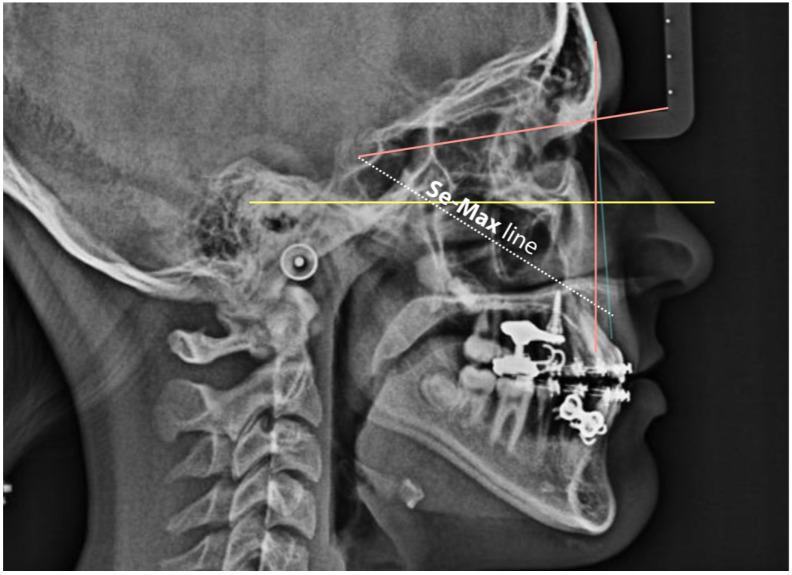
SeMax line, introduced by Arcos and Walter, was utilized to evaluate anteroposterior maxillary movement in a simple and reproducible manner. This parameter corresponds to the linear distance (in millimeters) between Sella (S) and point A.

In addition to angular and appraisal-based metrics, we used the SeMax line—a linear reference defined as the distance (mm) between Sella (S) and Point A (Figure 6). The SeMax line quantifies maxillary effective protraction (MEP), capturing forward skeletal displacement with reduced sensitivity to rotations or vertical changes compared with SNA. This measurement aligns with the natural craniofacial growth vector and sutural displacement—typically downward and forward—following cranial base and facial suture orientations [29,30].

### 2.7. Statistical Analysis

The normality of data distribution was assessed using the Shapiro–Wilk test. Effect sizes (Cohen’s d) were calculated to estimate the magnitude of treatment effects. Ninety-five percent confidence intervals (95% CI) were computed where appropriate.

Descriptive statistics summarized baseline and post-treatment values (mean ± SD). Normality of paired differences was verified; paired Student’s *t*-tests and Wilcoxon signed-rank tests compared pre- and post-treatment data. Significance was set at *p* < 0.05. Analyses were performed in SPSS v26 (IBM, Armonk, NY, USA). The Pearson correlation tested associations between the SeMax line (MEP) and SNA/Wits.

Given the pilot nature of the study, the statistical analysis was intended to explore trends and estimate the magnitude of effects, rather than to provide definitive confirmatory evidence.

## 3. Results

Ten patients (five males, five females) were analyzed (mean age 16.04 ± 1.28 years; late growth phase CS4–CS6). No relevant differences were observed between male and female patients; however, no statistical comparison was performed due to the limited sample size. Thirty cephalometric variables were computed per patient, including skeletal angular parameters, vertical relationships, incisor position/inclination, and linear indices (SeMax, Wits).

A significant increase in SNA was observed (76.9° ± 1.73 to 83.0° ± 1.76; *p* < 0.001). SNB showed no significant change (*p* = 0.897). L1’s position decreased (4.25 ± 1.16 to 3.56 ± 0.90 mm; *p* = 0.0158), whereas L1’s angulation increased by +2.0° without significance (*p* = 0.6744). U1’s position/angulation changes were minimal and nonsignificant. Overbite values were not consistently recorded in all patients and therefore were not included in the present analysis.

**Figure 7:**
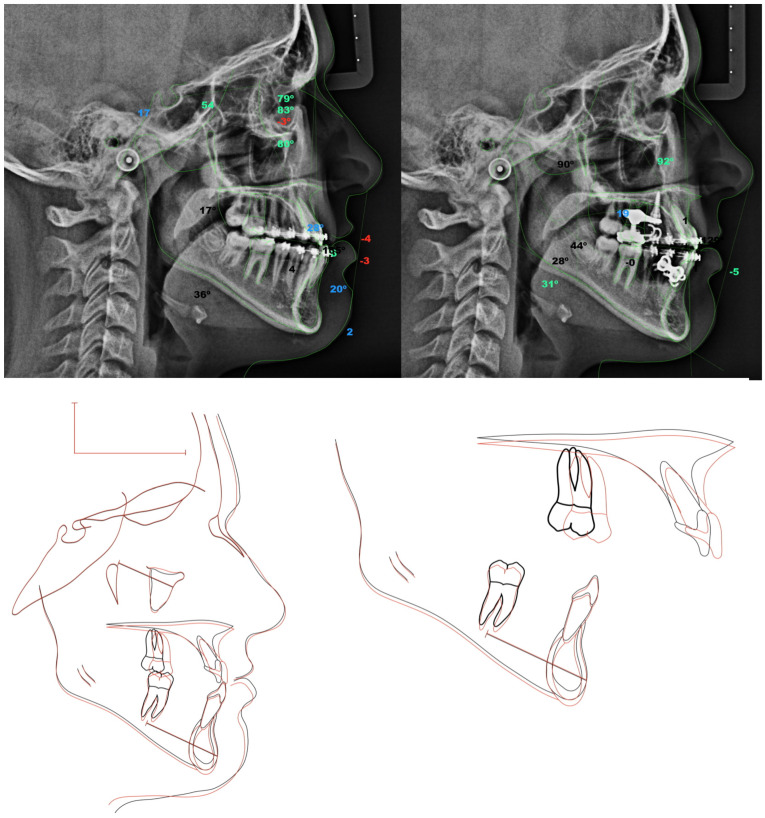
Cephalometric superimposition analysis (before and after orthopedic treatment).

**Figure 8:**
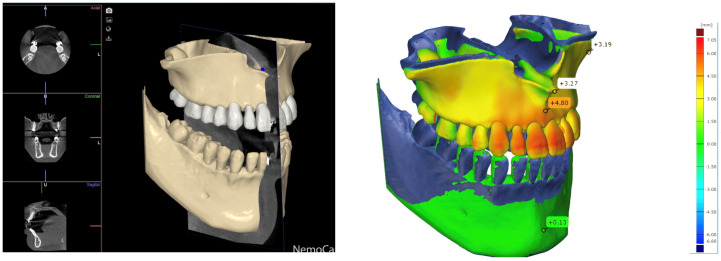
3D superimposition where the maxilla advancement effect is appreciated and measured.

Wits improved from −4.5 ± 2.3 to −1.2 ± 1.8 mm (*p* = 0.007). SeMax increased from 77.3 ± 1.9 to 80.2 ± 2.1 mm (*p* = 0.0013), consistent with the forward maxillary displacement (Table 1; Figure 7 and Figure 8).

Effect size analysis demonstrated large effects for maxillary advancement (SNA) and moderate-to-large effects for the Wits and SeMax changes, supporting the clinical relevance of the observed skeletal modifications.

### 3.1. Skeletal and Dental Effects

The significant increase in SNA (*p* < 0.001) indicates effective maxillary advancement. The SNB remained unchanged (*p* = 0.897), indicating mandibular stability. SN–GoGn increased slightly but not significantly (+0.7°, *p* = 0.328), suggesting preserved vertical control. Improvements in the Wits (+3.3 mm, *p* = 0.007) and SeMax (+2.9 mm, *p* = 0.0013) are consistent with sagittal correction and forward maxillary displacement. The lower incisor position decreased slightly (−0.69 mm; *p* = 0.0158) without significant angular change, and the upper incisors remained stable—consistent with minimal dentoalveolar side effects.

### 3.2. Correlation Analysis

Pearson correlations revealed weak but positive associations between SeMax and conventional sagittal measures: SeMax vs. SNA r = 0.349 (baseline) and r = 0.229 (post), SeMax vs. Wits r = 0.274 (pre) and r = 0.113 (post). These findings support SeMax as a distinct, rotation-resistant linear indicator of maxillary advancement (Table 2).

## 4. Discussion

The present pilot clinical study describes a digitally guided, relatively minimally invasive protocol compared to conventional subgingival miniplates and orthognathic surgery for skeletal Class III correction in late adolescents, and documents its pilot clinical and cephalometric outcomes. This approach integrates a bone-anchored hybrid maxillary expander with custom supragingival mandibular miniplates by using a fully digital workflow designed to enhance accuracy, reduce invasiveness, and improve clinical feasibility.

From a clinical perspective, the present protocol emphasizes reproducibility, relatively minimally invasive skeletal anchorage compared to conventional subgingival miniplates, and integration of digital planning into everyday orthodontic practice.

As a pilot clinical study, the primary aim was to document the feasibility and treatment response, rather than to provide definitive comparative evidence.

This study adds to the evidence that skeletal anchorage systems can produce clinically meaningful maxillary protraction in adolescents with limited dentoalveolar effects. Conventional RME/facemask protocols are compliance-dependent and often yield incisor proclination and clockwise mandibular rotation when used beyond early mixed dentition [15,17,31,32].

Using digitally guided supragingival mandibular miniplates with a hybrid maxillary expander and modified Alt-RAMEC, we observed significant increases in SNA and SeMax with stable SNB and minimal upper incisor change—indicating a predominantly skeletal response. Improved sagittal skeletal relationships may facilitate the functional adaptation of the stomatognathic system, promoting a more favorable neuromuscular balance and potentially contributing to improved functional stability beyond the observed cephalometric changes. These results are consistent with the bone-anchored protraction literature, including De Clerck et al. [33]. The main clinical advantage of this protocol lies in the combination of digitally guided miniscrew placement, a bone-anchored hybrid expander, and supragingival mandibular miniplates, allowing for continuous orthopedic traction with minimal dentoalveolar side effects and reduced surgical invasiveness compared to conventional subgingival miniplate systems. The digital workflow also facilitates accurate and reproducible placement of skeletal anchorage, potentially improving safety and clinical efficiency.

The magnitude of skeletal response at a mean age of 16 years suggests that bone-anchored traction—applied continuously and below the maxillary center of resistance—may broaden treatment possibilities in late adolescence [16]. Absent significant changes in SNB or SN–GoGn further indicate vertical control, contrasting with typical facemask effects [15].

Notably, the skeletal effects observed in this cohort occurred in patients in advanced stages of skeletal maturation (CS4–CS6), with a mean chronological age of approximately 16 years. In this study, “late adolescence” was defined according to skeletal maturation, rather than chronological age. This is particularly relevant, as conventional orthopedic protocols are typically age-dependent, whereas skeletal anchorage-based approaches may remain effective in patients approaching the end of active craniofacial growth. Therefore, the observed treatment effects should be interpreted in the context of skeletal maturity, rather than strict chronological age limits.

This finding is clinically relevant, as conventional facemask-based protocols typically show limited orthopedic effectiveness at this stage of development. To our knowledge, few clinical reports have described a fully digital workflow integrating guided miniscrew placement and custom supragingival mandibular plates for Class III orthopedic correction in late adolescence. This approach may expand treatment possibilities in patients approaching the end of active craniofacial growth who are not ideal candidates for early orthopedic intervention or who seek to avoid orthognathic surgery.

We also explored SeMax as a linear reference to quantify maxillary effective protraction. Compared with SNA and Wits—both influenced by cranial base or occlusal plane rotation—SeMax offers a rotation-resistant estimate of true sagittal advancement. Weak correlations with SNA and Wits (r ≈ 0.11–0.35) suggest complementary information and support its use as an outcome variable in future trials. The SeMax line was used as an exploratory complementary linear measurement to describe effective maxillary displacement, and further validation in controlled and three-dimensional studies is required before routine clinical adoption.

The SeMax line was used as an exploratory complementary linear parameter to describe effective maxillary displacement and should not be interpreted as a replacement for established cephalometric measurements.

Biomechanically, intermaxillary elastics anchored to palatal and supragingival mandibular plates provide continuous, physiologically oriented forces. The supragingival design reduces morbidity and chair time. The absence of screw failures is likely attributable to bicortical anchorage [34,35,36], precise guided placement with safe root clearance [37,38], and a plate design that minimizes soft-tissue interference and optimizes the load distribution [39].

The present study should be interpreted as a pilot clinical investigation aimed at describing a reproducible protocol and its preliminary outcomes.

## 5. Conclusions

Digitally guided supragingival mandibular miniplates combined with a hybrid maxillary expander and a modified Alt-RAMEC protocol may represent a promising, clinically feasible, relatively minimally invasive approach compared to conventional subgingival miniplates and orthognathic surgery for selected late adolescent patients with skeletal Class III malocclusion.

In this pilot cohort, the protocol was associated with significant maxillary advancement, limited dentoalveolar side effects, and stable mandibular and vertical parameters. These findings suggest that bone-anchored traction supported by digital planning may extend the therapeutic window for orthopedic correction beyond early adolescence. The SeMax line functioned as a complementary linear cephalometric parameter to the traditional angular and occlusal indicators.

## 6. Limitations and Future Directions

As a pilot clinical study, the present investigation should be interpreted in light of certain limitations. The small sample size (*n* = 10), the absence of a control group, and the lack of long-term retention data classify this investigation as a pilot clinical study. Therefore, the results should be interpreted cautiously and primarily as hypothesis-generating, rather than definitive evidence. Nevertheless, the observed skeletal effects suggest that supragingival miniplate-based orthopedic traction may represent a clinically feasible option in selected adolescent patients in whom conventional orthopedic approaches are limited. Future retrospective trials should define the optimal force magnitude and vectors, assess long-term skeletal stability, and further validate the SeMax line against three-dimensional outcomes. Residual growth effects cannot be excluded and may have contributed to part of the observed skeletal changes. Additionally, overbite was not consistently recorded and therefore could not be analyzed, which represents a limitation of the present study. 

## Figures and Tables

**Table 1:** Individual cephalometric measurements before and after treatment.

	P1	P2	P3	P4	P5	P6	P7	P8	P9	P10
**Age**	14.5	13.8	14.9	16.8	17.5	16.3	17.7	16.4	15.9	16.6
**Sex**	F	F	M	M	M	F	F	M	M	F
**Growth Pattern**	meso	dolico	meso	meso	meso	dolico	meso	dolico	meso	meso
**Initial SNA**	79	77	76	77	75	77	77	80	77	74
**Final SNA**	87	82	81	83	83	84	84	83	82	81
**SNA Difference**	−8	−5	−5	−6	−8	−7	−7	−3	−5	−7
**Initial SNB**	83	81	82	82	80	81	81.5	84	83	80
**Final SNB**	84	79	82.5	82.5	81	80	82	82	84	80
**SNB Difference**	−1	2	−0.5	−0.5	−2	1	−0.5	2	−1	0
**Initial ANB**	−4	−4	−6	−5	−5	−4	−4.5	−4	−6	−6
**Final ANB**	3	3	−1.5	0.5	3	4	2	1	−2	1
**ANB Difference**	−7	−7	−4.5	−5.5	−8	−8	−6.5	−5	−4	−7
**Initial SN-GoGn**	36	39	34	36	35	39	35	38	26	34
**Final SN-GoGn**	36	40	35	37	36.5	40.5	35	40	26	34
**SN-GoGn Difference**	0	−1	−1	−1	−1.5	−1.5	0	−2	0	0
**Pre-tx U1 Position**	5.6	6	4	3.7	2	4	6	5.5	5	3.5
**Post-tx U1 Position**	2.5	7	3	4	4	4	5.5	6	6.6	4.5
**U1 Position Difference**	3.1	−1	1	−0.3	−2	0	0.5	−0.5	−1.6	−1
**Pre-tx U1 Angulation**	28	24	26	25	27	25	31	26	18	20
**Post-tx U1 Angulation**	24	25	27	28	27	22	28	27	22	24
**U1 Angulation Difference**	4	−1	−1	−3	0	3	3	−1	−4	−4
**Pre-tx L1 Position**	3.8	5	4	1.7	3.5	6	4	5	4.5	5
**Post-tx L1 Position**	4.1	4	3	1.5	3.5	4	3	4.5	4.5	3.5
**L1 Position Difference**	−0.3	1	1	0.2	0	2	1	0.5	0	3.5
**Pre-tx L1 Angulation**	20	21	19	23	22	19	17	20	24	23
**Post-tx L1 Angulation**	22	23	23	24	23	22	24	21	24	24
**L1 Angulation Difference**	−2	−2	−4	−1	−1	−3	−7	−1	0	−1
**Pre-tx SeMax Line**	77.8	76	77	75.8	79	78	77.5	80	79	77
**Post-tx SeMax Line**	81	77.8	80.2	77.4	81.6	81.8	80.6	82.6	80.6	80.8
**SeMax Difference**	−3.2	−1.8	−3.2	−1.6	−2.6	−3.8	−3.1	−2.6	−1.6	−3.8
**Pre-tx Wits**	−9	−3	−5	−3.5	−1	−3	−3	−2	−1.8	−2
**Post-tx Wits**	−1.8	0	−2	1	1	1	2	1	2	2
**Vertebral Maturation**	CS4	CS 4	CS 4	CS 5	CS 5	CS5	CS6	CS 5	CS 4	CS 4

Values are expressed as mean ± standard deviation. Statistical significance was set at *p* < 0.05.

**Table 2:** Pearson correlations between SeMax line and conventional sagittal parameters.

Comparison	Correlation Coefficient (r)
SeMax vs. SNA (pre-treatment)	0.349
SeMax vs. SNA (post-treatment)	0.229
SeMax vs. Wits (pre-treatment)	0.274
SeMax vs. Wits (post-treatment)	0.113

## Data Availability

Data are available from the corresponding author upon reasonable request.

## References

[1] Guyer E.C., Ellis E.E., McNamara J.A., Behrents R.G. (1986). Components of Class III malocclusion in juveniles and adolescents. Angle Orthod..

[2] Ngan P.W., Hagg U., Yiu C., Wei S.H. (1997). Treatment response and long-term dentofacial adaptations to maxillary expansion and protraction. Semin. Orthod..

[3] Westwood P.V., McNamara J.A., Baccetti T., Franchi L., Sarver D.M. (2003). Long-term effects of Class III treatment with rapid maxillary expansion and facemask therapy. Am. J. Orthod. Dentofac. Orthop..

[4] Baccetti T., Franchi L., McNamara J.A. (2004). Cephalometric predictors of success/failure with RME + facemask. Am. J. Orthod. Dentofac. Orthop..

[5] Ngan P. (2006). Early treatment of Class III malocclusion: Is it worth the burden?. Am. J. Orthod. Dentofac. Orthop..

[6] Kim J.H., Viana M.A., Graber T.M., Omerza F.F., BeGole E.A. (1999). Effectiveness of protraction facemask: Meta-analysis. Am. J. Orthod. Dentofac. Orthop..

[7] Merwin D., Ngan P., Hagg U., Yiu C., Wei S.H. (1997). Timing for anteriorly directed orthopedic force to the maxilla. Am. J. Orthod. Dentofac. Orthop..

[8] Meireles R.A., Rocha J.E.T., Sampaio J.R.F., Sousa F.C.P., Coutinho H.D.M., Júnior R.F.F.P. (2025). Treatment of pattern III with maxillary protraction supported by mini-implants (MAMP technique). Pediatr. Dent. J..

[9] Wilmes B., Ngan P., Liou E.J., Franchi L., Drescher D. (2014). Hybrid-Hyrax and Alt-RAMEC. J. Clin. Orthod..

[10] De Clerck H.J., Cornelis M.A., Cevidanes L.H., Heymann G.C., Tulloch C.J. (2009). Maxillary traction with miniplates. J. Oral Maxillofac. Surg..

[11] Cevidanes L.H., Heymann G., Cornelis M.A., DeClerck H.J., Tulloch J.F. (2009). 3D CBCT superimposition in growing patients. Am. J. Orthod. Dentofac. Orthop..

[12] Choi S.H., Kang D.Y., Kim Y.H., Hwang C.J. (2014). Two-stage orthognathic surgery with mandibular step osteotomy. Am. J. Orthod. Dentofac. Orthop..

[13] Moon W. (2018). Facemask + MSE. Semin. Orthod..

[14] Nguyen T., Cevidanes L., Cornelis M.A., Heymann G., de Paula L.K., De Clerck H. (2011). 3D assessment with bone-anchored protraction. Am. J. Orthod. Dentofac. Orthop..

[15] Baccetti T., McGill J.S., Franchi L., McNamara J.A., Tollaro I. (1998). Early Class III treatment with RME/FM. Am. J. Orthod. Dentofac. Orthop..

[16] De Clerck H.J., Proffit W.R. (2015). Growth modification of the face: Class III. Am. J. Orthod. Dentofac. Orthop..

[17] Willmann J.H., Nienkemper M., Tarraf N.E., Wilmes B., Drescher D. (2018). Hybrid-Hyrax–Facemask vs Hybrid-Hyrax–Mentoplate. Prog. Orthod..

[18] Ronsivalle V., Venezia P., Bennici O., D’Antò V., Leonardi R., Giudice A.L. (2023). Accuracy of digital workflows for miniscrew placement. BMC Oral Health.

[19] Cassetta M., Altieri F., Di Giorgio R., Barbato E. (2018). CAD-CAM palatal miniscrew guide. Int. J. Oral Maxillofac. Surg..

[20] Winsauer H., Vlachojannis C., Bumann A., Vlachojannis J., Chrubasik S. (2014). Palatal bone height for mini-implant insertion. Eur. J. Orthod..

[21] Kravitz N.D., Kusnoto B. (2007). Risks and complications of orthodontic miniscrews. Am. J. Orthod. Dentofac. Orthop..

[22] Walter A., Winsauer H., Marcé-Nogué J., Mojal S., Puigdollers A. (2013). Mini-implant design, primary stability and fracture risk. Med. Oral Patol. Oral Cir. Bucal..

[23] Nguyen M.V., Codrington J., Fletcher L., Dreyer C.W., Sampson W.J. (2018). The influence of insertion torque. Eur. J. Orthod..

[24] Uchida Y., Namura Y., Motoyoshi M. (2023). Optimal insertion torque—Review. Appl. Sci..

[25] Masucci C., Franchi L., Giuntini V., Defraia E. (2014). Short-term effects of modified Alt-RAMEC. Orthod. Craniofac. Res..

[26] Liou E.J. (2005). Effective maxillary orthopedic protraction—Distraction analog. Prog. Orthod..

[27] Wang Y.C., Chang P.M., Liou E.J. (2009). Opening circumaxillary sutures with Alt-RAMEC. Angle Orthod..

[28] Petit H.P., McNamara J.A., Ribbens K.A., Howe R.P. (1983). Adaptation following accelerated facial mask therapy. Clinical Alterations of the Growing Face.

[29] Björk A. (1969). Prediction of mandibular growth rotation. Am. J. Orthod..

[30] Melsen B., Melsen F. (1982). Postnatal palatomaxillary development. Am. J. Orthod..

[31] Walter A., Lewington A.J., Iglesias A., Bousquet M., Auladell A. (2018). WB Late adolescence Class III correction with mini-implants + facemask. EC Dent. Sci..

[32] Franchi L., Statie M.D., Clauser T., Migliorati M., Ugolini A., Bucci R., Rongo R., Nucera R., Portelli M., McNamara J.A. (2025). Skeletal vs conventional anchorage: Delphi consensus. Prog. Orthod..

[33] De Clerck H.J., Nguyen T., de Paula L.K., Cevidanes L.H.S. (2012). Three-dimensional assessment of maxillary changes associated with bone-anchored maxillary protraction. Am. J. Orthod. Dentofac. Orthop..

[34] Brettin B.T., Grosland N.M., Qian F., Southard K.A., Stuntz T.D., Morgan T.A., Marshall S.D., Southard T.E. (2008). Bicortical vs monocortical anchorage. Am. J. Orthod. Dentofac. Orthop..

[35] Chen Y.J., Chang H.H., Huang C.Y., Hung H.C., Lai E.H., Yao C.C. (2007). Failure rate of skeletal anchorage systems. Clin. Oral Implant. Res..

[36] Marcé-Nogué J., Walter A., Gil L., Puigdollers A. (2013). Finite element comparison of orthodontic microscrews. Int. J. Oral Maxillofac. Implant..

[37] Choi E.A., Kim D., Jing L., Yu H.S., Choi S.H., Cha J.Y. (2025). Miniscrew surgical guide, success and root proximity. Korean J. Orthod..

[38] Asscherickx K., Vande Vannet B., Wehrbein H., Sabzevar M.M. (2008). Success rate relative to root proximity. Eur. J. Orthod..

[39] Leung M.T., Rabie A.B., Wong R.W. (2008). Stability of connected mini-implants and miniplates. Eur. J. Orthod..

